# Antiplatelet therapy in acute myocardial infarction complicated by cardiogenic shock

**DOI:** 10.1007/s00392-025-02619-2

**Published:** 2025-03-04

**Authors:** Angela Dettling, Kurt Huber, Steen D. Kristensen, Daniel Aradi, Benedikt Schrage, Peter Clemmensen

**Affiliations:** 1 Department of Cardiology, University Medical Center Hamburg-Eppendorf, Martinistr. 52, 20246 Hamburg, Germany; 2 German Center for Cardiovascular Research (DZHK), Partner Site Hamburg/Kiel/Lübeck, Hamburg, Germany; 3 Center for Population Health Innovation, POINT, Hamburg, Germany; 4 Faculty of Medicine, Sigmund Freud University, Vienna, Austria; 5 Department of Cardiology, Department of Clinical Medicine, Aarhus University Hospital and Aarhus University, Aarhus, Denmark; 6 Heart and Vascular Center, Semmelweis University, Budapest, Hungary

**Keywords:** Dual antiplatelet therapy, Antithrombotic therapy, Acute myocardial infarction, Cardiogenic shock, Mechanical circulatory support

## Abstract

Coronary revascularization represents a cornerstone in the treatment of infarct-related cardiogenic shock (CS). Early and effective antithrombotic therapy is critical and has been shown to improve mortality in most patients with acute coronary syndrome. Achieving early effective platelet inhibition and anticoagulation, with minimal risk, is particularly important in those high-risk patients with CS as the mortality remains high at approximately 50%. However, patients with CS are at high risk for both early thrombotic as well as bleeding events and striking the right balance remains a challenge due to a multitude of factors related to drug administration, metabolism and mechanical issues related to therapeutic interventions such as increasing use of mechanical circulatory support (MCS). This review therefore aims to provide an overview of the current practice, the underlying challenges and existing evidence on safety, efficacy and outcomes of adjunctive antiplatelet and antithrombotic therapy in patients with acute myocardial infarction (AMI) complicated by CS and discusses the use of parenteral platelet inhibitors.

## Introduction

Cardiogenic shock (CS) complicating acute myocardial infarction (AMI) occurs in 5–10% of all AMI patients and is associated with considerable worsening of the prognosis [1, 2]. Despite significant advances in cardiovascular medicine, the mortality associated with CS is remaining high at approximately 50% at 30 days [3, 4]. A randomized study showed that patients with AMI complicated by CS should undergo primary percutaneous coronary intervention (PCI) with intervention of the culprit lesion only as it is associated with a better outcome than early complete revascularization [5]. Hopes have been set in mechanical circulatory support devices (MCS) to improve outcomes of CS by restoring cardiac output. However, evidence of survival benefit in comparison to conventional care is still missing. The few randomized controlled trials (RCT) addressing MCS have shown neutral results for insertion of intra-aortic balloon pumps [6] as well as extra-corporeal life support (ECLS) [7], which requires large bore size for access. Recently, the DANGER Shock trial [8] offered a positive survival outcome after 6 months in a special cohort of patients suffering from AMI-related CS. Patients included suffered from typical ST-elevation myocardial infarction (STEMI), hypotension and hypoperfusion, had a left ventricular (LV) ejection fraction of < 45% and were randomized as soon as shock was diagnosed. Exclusion criteria were comatose patients with out-of-hospital cardiac arrest (OHCA), patients with right ventricle MI and such with AMI-related mechanical complications. Only every third patient with AMI-related CS belongs to this specific patient cohort [9]. The adjunctive medical therapy for these devices includes anticoagulation with heparins during and sometimes after the intervention and antiplatelet therapy which often is dual (DAPT) consisting of aspirin and a P2Y_12_-receptor inhibitor in patients with AMI-CS undergoing primary PCI [10].

In AMI patients, undergoing PCI early and timely DAPT has been shown to improve mortality and stent thrombosis rates [11, 12]. However, CS was an exclusion criterion in almost all the pivotal AMI trials investigating concomitant adjunctive DAPT [11, 12]. Mostly because patients with AMI complicated by CS present multiple challenges specifically related to the administration, metabolism and safety of DAPT, but also because other therapeutic interventions used in CS, such as MCS, increase the risk of bleeding and other complications [13–16]. Consequently, the medical management of patients with AMI-CS is much less driven by evidence-based recommendations compared to the overall AMI population. This review therefore aims to provide an overview of the current practice, the underlying challenges and existing evidence on safety, efficacy and outcomes of adjunctive DAPT in patients with AMI complicated by CS and to discuss the use of parenteral platelet inhibitors.

## Current practice and guidelines regarding adjunctive antiplatelet therapy in patients with AMI-CS

Achieving effective platelet inhibition with minimal risk of bleeding is particularly important in high-risk AMI patients undergoing complex PCI which is often the case in patients with CS. However, prospective randomized data are not available for antiplatelet treatment in AMI-CS as the pivotal trials comparing the more potent P2Y_12_-receptor inhibitors TRITON-TIMI 38 (prasugrel) [11] and PLATO (ticagrelor) [12] did not cover the subpopulation of patients with AMI-CS. To date, treatment guidelines are mostly adopted from these non-shock AMI trials [10]. However, data from randomized trials and registries imply large differences in the use of adjunctive DAPT after PCI in patients with AMI-CS (Table 1). The predominantly used drug is clopidogrel although the use of prasugrel and ticagrelor has increased [17].

**Table 1:**
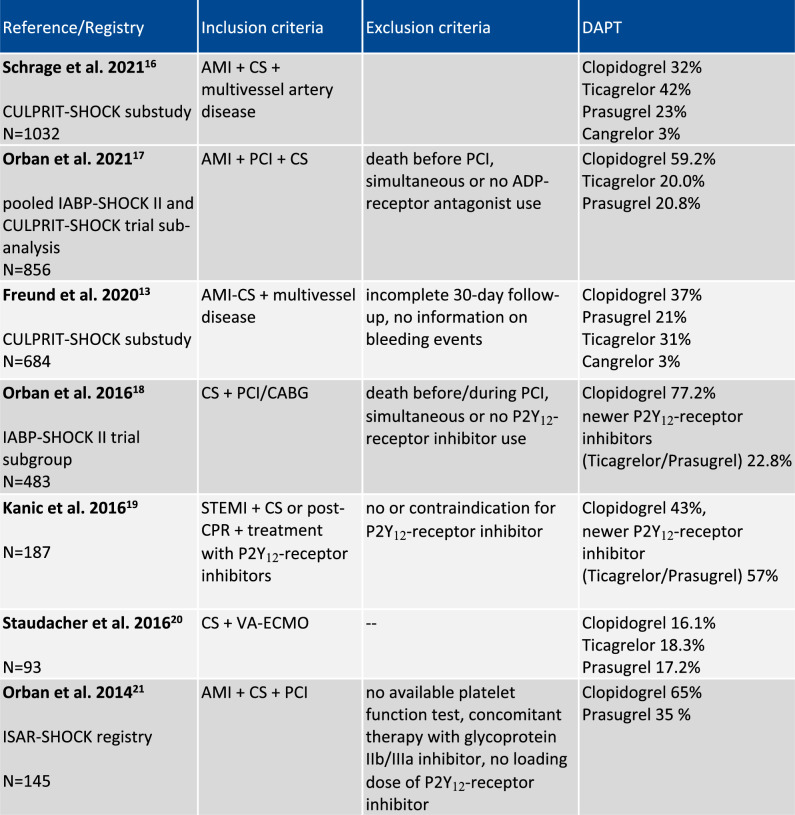
Distribution of dual antiplatelet therapy (DAPT) in current AMI-CS trials

Light gray indicates the predominant use of clopidogrel. Dark gray indicates the predominant use of newer P2Y_12_-receptor inhibitors (ticagrelor and prasugrel)

*AMI* acute myocardial infarction, *CS* cardiogenic shock, *PCI* percutaneous coronary intervention, *CABG* coronary artery bypass graft, *CPR* Cardiopulmonary resuscitation, *VA-ECMO* veno-arterial extra-corporeal membrane oxygenation

**Fig. 1:**
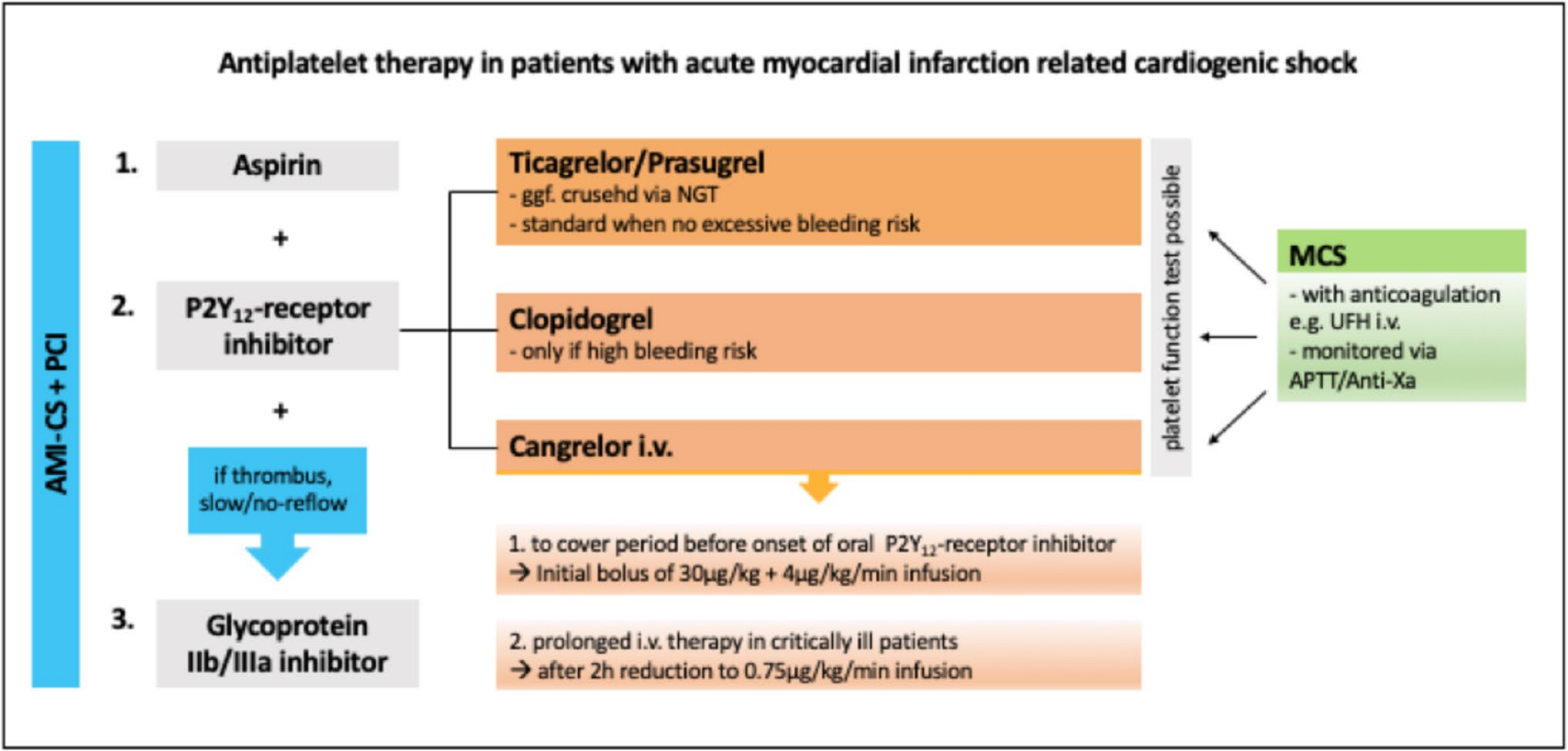
Current guidelines of dual antiplatelet therapy (DAPT) in patients with acute myocardial infarction-related cardiogenic shock (AMI-CS). *PCI* percutaneous coronary intervention, *MCS* mechanical circulatory support, *I.v.* intravenously. (Modified after Gorog et al. [10])

A recently published position paper from the European Society of Cardiology (ESC) outlined the current treatment guidelines to optimize and harmonize DAPT in AMI complicated by CS or OHCA (Fig. 1) [10]. In uncomplicated CS, the DAPT strategy suggestion is identical to non-shock AMI. Prasugrel and ticagrelor should be used when there is no excessive bleeding risk, whereas clopidogrel should be the drug of choice in patients with high bleeding risk (e.g., recent intracranial or gastrointestinal bleeding). The application of crushed prasugrel and ticagrelor through a nasogastric tube (NGT) allows a faster onset of platelet inhibition. Alternatively, and with an even much faster optimal platelet inhibition, a parenteral antiplatelet therapy using cangrelor is recommended to cover the period before onset of oral P2Y_12_-receptor inhibitors, and are used especially in comatose patients. Glycoprotein IIb/IIIa inhibitors should be considered as a bailout therapy in patients with angiographic evidence of large thrombus, slow or no-reflow and other thrombotic complications.

Cangrelor is a reversible P2Y_12_-receptor inhibitor, which possesses ideal pharmacokinetic properties because of its rapid onset and short half-life (3–6 min) with return of platelet activity within one hour [24]. The use of cangrelor was introduced in the 3 phase 3 Cangrelor Versus Standard Therapy to Achieve Optimal Management of Platelet Inhibition (CHAMPION) trials supporting its utility in contemporary PCI. Cangrelor treatment compared with clopidogrel reduced periprocedural ischemic complications while only an increased risk of Global Use of Strategies to Open Occluded Coronary Artery (GUSTO) mild bleeding was observed [25]. Since European and FDA approval in 2015 cangrelor is currently administered in a higher-risk cohort than represented in the CHAMPION program, most commonly in the context of PCI for STEMI and often with left main PCI, thrombus aspiration and cardiac arrest (CA). However, early observational studies suggest that cangrelor is well tolerated and associated with low rates of clinically significant ischemic or bleeding events [26–28]. Although, not formally tested in CS, initial results seem to support the potential role of cangrelor in this setting [29–32]. The standard administration of cangrelor consists of an intravenous bolus dose of 30 µg/kg followed by a 4 µg/kg/min infusion. For transition to an oral P2Y_12_-inhibitor, most commonly ticagrelor is being used [26, 33, 34].

Critically ill patients with CS after or during PCI may require MCS via veno-arterial extra-corporeal membrane oxygenation (VA-ECMO) and/or Impella® in order to restore hemodynamic stability. To avoid clotting of the circuit and reduce risk of embolization, anticoagulation is required as long as the MCS is in place. This exposes patients to increased risk of bleeding complications which may increase mortality. Anticoagulation is generally achieved with unfractionated heparin (UFH) and monitored via activated partial thromboplastin time (APTT). Because of a complex activation of both thrombosis and bleeding in MCS supported critically ill patients, a parallel anti-Factor Xa/APTT-guided anticoagulation algorithm has also been suggested in an attempt to optimize safety [35]. Additional DAPT is essential with a current recommendation of aspirin and clopidogrel. Cangrelor can also be considered for an extended period in addition to the anticoagulation. In case of prolonged infusion, a lower infusion dose often is used (after standard dose of 4 µg/kg/min, decrease to 0.75 µg/kg/min infusion after two hours until removal) [36]. The ability of cangrelor to promptly influence platelet activity in a titratable, dose-dependent manner makes it an attractive agent in critically ill patients on MCS. However, in patients who have received prolonged cardiac massage, caution should be undertaken to avoid serious bleeding [28].

## Unique challenges of adjunctive DAPT in patients with CS

**Fig. 2:**
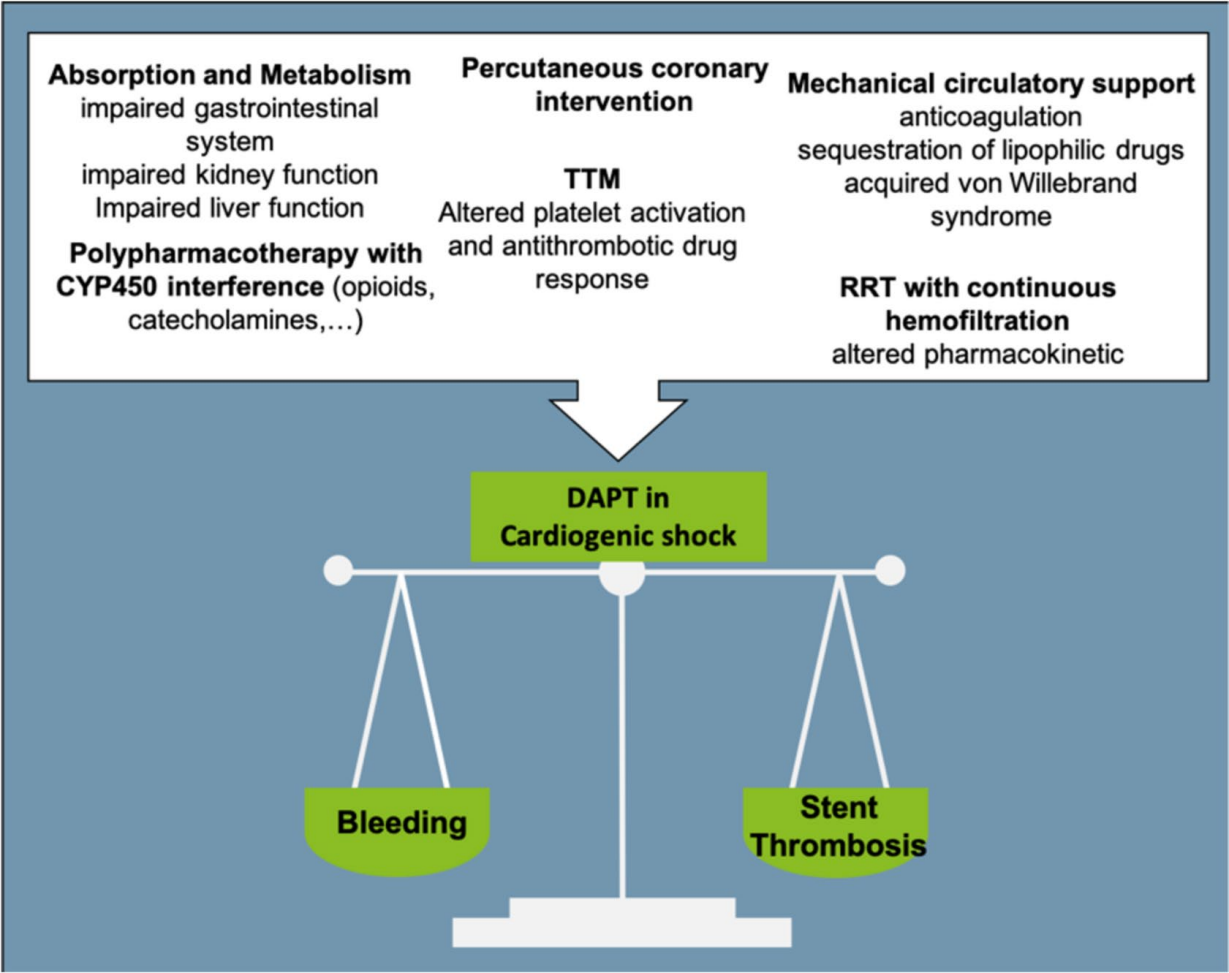
Factors influencing dual antiplatelet therapy (DAPT) in patients with acute myocardial infarction complicated by cardiogenic shock. *RRT* renal replacement therapy, *TTM* targeted temperature management

There are many unique challenges associated with achieving effective (i.e., avoid stent thrombosis) and safe (i.e., minimize bleeding events) antithrombotic therapy with DAPT in patients with CS (Fig. 2). First, CS has a profound effect on drug absorption and metabolism. Most antiplatelet agents are administered orally, absorbed gastrointestinally, may require hepatic activation and are excreted renally [37]. Both clopidogrel and prasugrel are prodrugs necessitating liver metabolism to deliver its active metabolite into the systemic circulation. Due to disturbance of the microcirculation, reduced gastrointestinal blood flow, frequent restriction of hepatic, and renal function in shock absorption and activation might be reduced and half-life increased [38, 39]. Second, additional polypharmacy including the use of opioids and catecholamines with possible interference of the cytochrome p450-dependent metabolism results in slower onset of platelet inhibition for all three oral P2Y_12_-receptor inhibitors [13, 40, 41]. Clopidogrel is known to be associated with a relatively slow onset of action and to induce lower levels of platelet inhibition in patients with AMI complicated by CS [16]. Third, therapeutic interventions such as targeted temperature management (TTM) sometimes used in patients after CA and especially affecting the inhibitory effect of clopidogrel, renal replacement therapy and MCS are also known to influence the antiplatelet efficacy of various P2Y_12_-receptor inhibitors [14, 16, 42–44]. Therefore, the effect of DAPT can only be predicted to a limited extent leading to an increased and unclear risk of stent thrombosis and bleeding in patients with CS.

To address the delayed onset and reduced antiplatelet effects of orally administered P2Y_12_-receptor inhibitors, various strategies have been explored [45, 46]. One possibility is the application of crushed tablets via a NGT [46]. Studies have shown that all three P2Y_12_-receptor inhibitors—clopidogrel, ticagrelor, and prasugrel—exhibit faster and greater bioavailability when administered in this manner: The initial studies evaluating the pharmacodynamics and kinetics of crushed clopidogrel tablets emerged around 2009 [47]. Subsequently, the MOJITO trial in 2015 demonstrated that administering crushed ticagrelor tablets to STEMI patients results in earlier platelet inhibition compared to standard tablets [48]. Similarly, the CRUSH Study in 2016 found that crushed prasugrel tablets administered to STEMI patients provided enhanced platelet inhibitory effects compared to whole tablets, especially in the initial hours after drug administration [49]. The absorption was threefold faster and a reduced platelet reactivity was observed as early as 30 min after drug administration with crushed prasugrel compared to whole tablets. Further studies have confirmed these findings, highlighting the increased speed of onset with crushed applications via NGT [50, 51]. However, this method does not entirely bridge the gap in delay in platelet inhibition, and there is still a need for large randomized trials, particularly in CS patients, to assess clinical endpoints.

Cangrelor, the intravenous P2Y_12_-receptor inhibitor mentioned above, requires no bioactivation in vivo [24], and might therefore pose a suitable alternative. Given the intravenous route of administration, cangrelor also circumvents any concerns of slow absorption and can overcome challenges associated with enteral access in intubated patients. It is currently under investigation in the DAPT-SHOCK-AMI (NCT03551964) trial, evaluating the effects of cangrelor versus crushed ticagrelor in CS patients undergoing PCI, focusing on a primary combined endpoint of death, myocardial infarction, and stroke. The use of cangrelor is supported by promising results in a multicenter registry [29].

The randomized IABP-SHOCK II trial reported a bleeding incidence of about 20% in infarct-related CS, a 2- to tenfold increase compared to AMI patients without shock [6]. In a sub-analysis of the CULPRIT-SHOCK trial, Freund et al. confirmed the frequency of bleeding in AMI-CS being remarkably higher compared to AMI patients without CS [15]. Furthermore, they identified VA-ECMO or Impella® treatment as a significant predictor of bleeding. Patients with bleeding events were associated with a significantly higher 30-day mortality. Accordingly, Schrage et al. could demonstrate an increase in life-threatening bleeding and peripheral vascular complications in patients with AMI-CS treated with Impella [52]. One explanation could be too intense anticoagulation as well as a low platelet count [53] which is often seen in CS and can exacerbate bleeding when using MCS devices [54]. Patients also frequently develop acquired von Willebrand factor defect within 24 h of ECMO implantation which significantly increases bleeding risk [55]. Moreover, ECMO circuits may induce sequestration of lipophilic drugs, increase the volume of distribution and decrease drug clearance [44].

## Current evidence of DAPT in CS regarding safety, efficacy, and outcomes

The optimization of adjunctive DAPT therapy in patients with AMI-CS represents an evolving aspect of cardiac critical care. The available data that complement the evidence outlined in non-shock AMI trials are limited to predominantly retrospective cohorts and observational studies [18]. Generally, outcomes were assessed based on mortality rate, stent thrombosis and bleeding events and are showing conflicting results: A recent pooled sub-analysis of the IABP-SHOCK II and CULPRIT-SHOCK trial comparing all three oral P2Y_12_-receptor inhibitors showed no significant difference in one-year mortality in patients treated with prasugrel vs. clopidogrel and ticagrelor vs. clopidogrel. Also, there was no increase in moderate or severe in-hospital or one-year bleeding events using prasugrel or ticagrelor vs. clopidogrel. In fact, the rates of moderate or severe in-hospital and one-year bleeding events were significantly lower in patients treated with ticagrelor than in patients treated with clopidogrel [19]. And in previous studies newer P2Y_12_-receptor inhibitors (prasugrel or ticagrelor) were associated with lower mortality in AMI-CS with no significant differences in observed bleeding events and stent thrombosis when compared to clopidogrel [20, 23]. Furthermore, in a retrospective analysis of STEMI patients presenting with CS and/or CA, the administration of newer P2Y_12_-receptor inhibitors (prasugrel or ticagrelor) was associated with a reduced one-year mortality in comparison to clopidogrel [21]. Regarding bleeding events and stent thrombosis, no differences between the P2Y_12_-receptor inhibitors were observed. Moreover, in line with a recent metaanalysis by Potalla et al. including studies of patients with AMI-CS receiving DAPT, the newer, more potent P2Y_12_-receptor inhibitors were associated with lower rates of early and one-year mortality with no significant differences in bleeding events [17]. In summary, these studies support the use of the potent newer P2Y1_12_-receptor inhibitors (prasugrel or ticagrelor) and provide some assurance regarding their safety in CS.

The available data evaluating the novel and use of cangrelor in AMI-CS are even more scarce. A retrospective study by Droppa et al. compared patients with AMI-CS treated with cangrelor to patients from the IABP-SHOCK II trial not receiving cangrelor (i.e., oral P2Y_12_-receptor inhibitors). Within 12-months of follow-up, the use of cangrelor was associated with greater improvement in TIMI-flow during PCI compared to oral P2Y_12_-receptor inhibitors and similar risk of bleeding events, stent thrombosis and mortality [31]. Moreover, recently, first results of the multicenter CAN-SHOCK trial were published [29]. This retrospective study compared the use of cangrelor in high-risk AMI patients after CA or with CS to matched patients of the CULPRIT-SHOCK trial. Overall cangrelor was associated with a low rate of stent thrombosis and reinfarction within the first 48 h after PCI. The reported number of ischemic and bleeding events was numerically lower albeit not statistically significant compared to the control. These results are in line with previous findings of the CANGRELOR-OHCA study in comatose OHCA patients undergoing PCI and TTM [30] and reported real-world experience with cangrelor in patients with CS [32], implying that cangrelor might offer a potentially safe and effective antiplatelet option.

## Current evidence of DAPT in patients with CS during MCS

The competing thrombotic and bleeding risk management during MCS remains challenging especially when a combination of DAPT and anticoagulation is required [56]. To date, only smaller studies showing partially conflicting results are available. Hence, the impact of efficacy and the safety of antithrombotic drugs in addition to anticoagulation in patients treated with MCS remain largely unclear. A retrospective study of Staudacher et al. including 93 patients on VA-ECMO support showed no difference in bleeding incidence in patients with DAPT in addition to UFH when compared to those without DAPT [22]. Also, the rate of transfusion of red blood cells was similar in patients with or without DAPT. Contrary to these findings, Iskaros et al. identified DAPT as an independent risk factor for bleeding in patients under treatment with Impella® support [57].

The clinical use of cangrelor during MCS following PCI has only been evaluated in small case series: Katz et al. reported a case series of 17 patients on VA-ECMO and/or Impella® support treated a with triple antithrombotic therapy regimen consisting of aspirin, cangrelor, and UFH. During a 30-day in-hospital follow-up, 59% of these patients experienced a bleeding event, of which 70% were classified as major. No patient experienced stent thrombosis [58]. Ciolek et al. reported a small case series of 13 patients, with similar antithrombotic treatment of which 77% of patients experienced a bleeding event [59]. In the studies described above, bleeding risk in patients with MCS and DAPT was 60% [22].

## Conclusion

Urgent culprit artery PCI in patients with AMI-CS remain the cornerstone among the current treatment options. The guidelines regarding DAPT therapy, during and after PCI are based on the results from non-shock AMI trials, leaving the CS cohort understudied and the optimal antiplatelet therapy less well understood. This review demonstrates the lack of consistency regarding DAPT in patients with CS undergoing PCI. This could be critical as patients with CS are at high risk for both early thrombotic as well as bleeding events. Parental antiplatelet agents should currently be a choice of treatment in AMI-CS patients due to their fast antiplatelet activity that does not depend on enteral access and adsorption. The lack of evidence-based treatment protocols regarding antithrombotic therapy in the AMI-CS population could potentially contribute to the high mortality that has been unchanged for decades. To develop sufficient treatment recommendations, accelerated research in this field is needed to answer the unresolved issues, first and foremost the question of optimal DAPT strategy in patients with CS, especially in those treated with MCS.

## Abbreviations

AMI: Acute myocardial infarction
APTT: Activated partial thromboplastin time
CA: Cardiac arrest
CS: Cardiogenic shock
DAPT: Dual antiplatelet therapy
ECLS: Extra-corporeal life support
MCS: Mechanical circulatory support
NGT: Nasogastric tube
OHCA: Out-of-hospital cardiac arrest
PCI: Percutaneous coronary intervention
TTM: Targeted temperature management
UFH: Unfractionated heparin
VA-ECMO: Veno-arterial extra-corporeal membrane oxygenation therapy
